# Supplementary material for: Unveiling the Bioactive Potential of the Invasive Jellyfish Phyllorhiza punctata Through Integrative Transcriptomic and Proteomic Analyses
Source: Biomolecules. 2025 Aug 4;15(8):1121. doi: 10.3390/biom15081121 (PMC12383608; doi:10.3390/biom15081121)
Supplement: Supplementary file 1 [file biomolecules-15-01121-s001.zip › Suplementary Material/FileS3/PEPFOLD4/XP_065061103_11376/clusters.html]

 PEP-FOLD clusters


Download archive of all models  here   

|  |  |  |
| --- | --- | --- |
| # | file | sOPEP |
| # cluster 1 - Size: 8 |  |  |
|  | XP\_065061103\_11376-model1 | -19.1447 |
|  | XP\_065061103\_11376-model1.1 | -19.0056 |
|  | XP\_065061103\_11376-model1.2 | -18.996 |
|  | XP\_065061103\_11376-model1.3 | -18.9753 |
|  | XP\_065061103\_11376-model1.4 | -18.9753 |
|  | XP\_065061103\_11376-model1.5 | -18.9753 |
|  | XP\_065061103\_11376-model1.6 | -18.6602 |
|  | XP\_065061103\_11376-model1.7 | -18.6096 |
| # cluster 2 - Size: 18 |  |  |
|  | XP\_065061103\_11376-model2 | -19.0438 |
|  | XP\_065061103\_11376-model2.1 | -19.0438 |
|  | XP\_065061103\_11376-model2.2 | -19.0438 |
|  | XP\_065061103\_11376-model2.3 | -19.0438 |
|  | XP\_065061103\_11376-model2.4 | -19.0438 |
|  | XP\_065061103\_11376-model2.5 | -19.0438 |
|  | XP\_065061103\_11376-model2.6 | -19.0438 |
|  | XP\_065061103\_11376-model2.7 | -19.0438 |
|  | XP\_065061103\_11376-model2.8 | -19.0438 |
|  | XP\_065061103\_11376-model2.9 | -19.0438 |
|  | XP\_065061103\_11376-model2.10 | -19.0438 |
|  | XP\_065061103\_11376-model2.11 | -18.9028 |
|  | XP\_065061103\_11376-model2.12 | -18.8236 |
|  | XP\_065061103\_11376-model2.13 | -18.7724 |
|  | XP\_065061103\_11376-model2.14 | -18.7724 |
|  | XP\_065061103\_11376-model2.15 | -18.7724 |
|  | XP\_065061103\_11376-model2.16 | -18.6441 |
|  | XP\_065061103\_11376-model2.17 | -18.5912 |
| # cluster 3 - Size: 9 |  |  |
|  | XP\_065061103\_11376-model3 | -19.0126 |
|  | XP\_065061103\_11376-model3.1 | -19.0126 |
|  | XP\_065061103\_11376-model3.2 | -18.9208 |
|  | XP\_065061103\_11376-model3.3 | -18.9208 |
|  | XP\_065061103\_11376-model3.4 | -18.9208 |
|  | XP\_065061103\_11376-model3.5 | -18.8899 |
|  | XP\_065061103\_11376-model3.6 | -18.8759 |
|  | XP\_065061103\_11376-model3.7 | -18.6802 |
|  | XP\_065061103\_11376-model3.8 | -18.5262 |
| # cluster 4 - Size: 7 |  |  |
|  | XP\_065061103\_11376-model4 | -18.9876 |
|  | XP\_065061103\_11376-model4.1 | -18.9876 |
|  | XP\_065061103\_11376-model4.2 | -18.9876 |
|  | XP\_065061103\_11376-model4.3 | -18.9876 |
|  | XP\_065061103\_11376-model4.4 | -18.8479 |
|  | XP\_065061103\_11376-model4.5 | -18.8016 |
|  | XP\_065061103\_11376-model4.6 | -18.6265 |
| # cluster 5 - Size: 3 |  |  |
|  | XP\_065061103\_11376-model5 | -18.9662 |
|  | XP\_065061103\_11376-model5.1 | -18.6781 |
|  | XP\_065061103\_11376-model5.2 | -18.626 |
| # cluster 6 - Size: 9 |  |  |
|  | XP\_065061103\_11376-model6 | -18.9367 |
|  | XP\_065061103\_11376-model6.1 | -18.9367 |
|  | XP\_065061103\_11376-model6.2 | -18.9367 |
|  | XP\_065061103\_11376-model6.3 | -18.9367 |
|  | XP\_065061103\_11376-model6.4 | -18.9367 |
|  | XP\_065061103\_11376-model6.5 | -18.9367 |
|  | XP\_065061103\_11376-model6.6 | -18.9367 |
|  | XP\_065061103\_11376-model6.7 | -18.6988 |
|  | XP\_065061103\_11376-model6.8 | -18.56 |
| # cluster 7 - Size: 2 |  |  |
|  | XP\_065061103\_11376-model7 | -18.8755 |
|  | XP\_065061103\_11376-model7.1 | -18.7711 |
| # cluster 8 - Size: 2 |  |  |
|  | XP\_065061103\_11376-model8 | -18.8358 |
|  | XP\_065061103\_11376-model8.1 | -18.7898 |
| # cluster 9 - Size: 5 |  |  |
|  | XP\_065061103\_11376-model9 | -18.8207 |
|  | XP\_065061103\_11376-model9.1 | -18.7591 |
|  | XP\_065061103\_11376-model9.2 | -18.7157 |
|  | XP\_065061103\_11376-model9.3 | -18.7153 |
|  | XP\_065061103\_11376-model9.4 | -18.4829 |
| # cluster 10 - Size: 4 |  |  |
|  | XP\_065061103\_11376-model10 | -18.8168 |
|  | XP\_065061103\_11376-model10.1 | -18.8163 |
|  | XP\_065061103\_11376-model10.2 | -18.7918 |
|  | XP\_065061103\_11376-model10.3 | -18.7813 |
| # cluster 11 - Size: 5 |  |  |
|  | XP\_065061103\_11376-model11 | -18.7949 |
|  | XP\_065061103\_11376-model11.1 | -18.7168 |
|  | XP\_065061103\_11376-model11.2 | -18.7168 |
|  | XP\_065061103\_11376-model11.3 | -18.7168 |
|  | XP\_065061103\_11376-model11.4 | -18.6756 |
| # cluster 12 - Size: 3 |  |  |
|  | XP\_065061103\_11376-model12 | -18.783 |
|  | XP\_065061103\_11376-model12.1 | -18.783 |
|  | XP\_065061103\_11376-model12.2 | -18.7362 |
| # cluster 13 - Size: 5 |  |  |
|  | XP\_065061103\_11376-model13 | -18.7701 |
|  | XP\_065061103\_11376-model13.1 | -18.6998 |
|  | XP\_065061103\_11376-model13.2 | -18.6482 |
|  | XP\_065061103\_11376-model13.3 | -18.6482 |
|  | XP\_065061103\_11376-model13.4 | -18.5671 |
| # cluster 14 - Size: 4 |  |  |
|  | XP\_065061103\_11376-model14 | -18.7477 |
|  | XP\_065061103\_11376-model14.1 | -18.7129 |
|  | XP\_065061103\_11376-model14.2 | -18.7129 |
|  | XP\_065061103\_11376-model14.3 | -18.6985 |
| # cluster 15 - Size: 1 |  |  |
|  | XP\_065061103\_11376-model15 | -18.7294 |
| # cluster 16 - Size: 2 |  |  |
|  | XP\_065061103\_11376-model16 | -18.7196 |
|  | XP\_065061103\_11376-model16.1 | -18.5147 |
| # cluster 17 - Size: 1 |  |  |
|  | XP\_065061103\_11376-model17 | -18.7119 |
| # cluster 18 - Size: 9 |  |  |
|  | XP\_065061103\_11376-model18 | -18.6993 |
|  | XP\_065061103\_11376-model18.1 | -18.6993 |
|  | XP\_065061103\_11376-model18.2 | -18.6993 |
|  | XP\_065061103\_11376-model18.3 | -18.6993 |
|  | XP\_065061103\_11376-model18.4 | -18.661 |
|  | XP\_065061103\_11376-model18.5 | -18.5997 |
|  | XP\_065061103\_11376-model18.6 | -18.5997 |
|  | XP\_065061103\_11376-model18.7 | -18.5408 |
|  | XP\_065061103\_11376-model18.8 | -18.5291 |
| # cluster 19 - Size: 2 |  |  |
|  | XP\_065061103\_11376-model19 | -18.6947 |
|  | XP\_065061103\_11376-model19.1 | -18.5951 |
| # cluster 20 - Size: 1 |  |  |
|  | XP\_065061103\_11376-model20 | -18.5636 |
|  |  |  |
